# Academic Performance Among Children With Sickle Cell Disease in Low‐ and Middle‐Income Countries: A Systematic Review

**DOI:** 10.1002/hsr2.73150

**Published:** 2026-09-03

**Authors:** Shubaya K. Naggayi, Paul Bangirana, Simple Ouma, Mary Nyakato, Alison A. Kinengyere, Dickens Akena, Nancy S. Green, Richard Idro

**Affiliations:** ^1^ Department of Paediatrics and Child Health Makerere University College of Health Sciences Kampala Uganda; ^2^ Global Health Uganda Kampala Uganda; ^3^ Department of Psychiatry Makerere University College of Health Sciences Kampala Uganda; ^4^ Department of Public Health Soroti University Soroti Uganda; ^5^ Sir Albert Cook Library Makerere University College of Health Sciences Kampala Uganda; ^6^ Africa Center for Systematic Reviews & Knowledge Translation Makerere University College of Health Sciences Kampala Uganda; ^7^ Department of Pediatrics, Division of Hematology, Oncology and Stem Cell Transplantation Columbia University Irving Medical Center New York USA

**Keywords:** academic performance, anemia, child, developing countries, sickle cell

## Abstract

**Background and Aim:**

Children with sickle cell disease (SCD) in low‐ and middle‐income countries (LMICs) face significant disease‐related challenges and socioeconomic status (SES) disparities that may negatively impact their academic performance. This systematic review synthesized existing evidence on academic performance among children with SCD in LMICs.

**Methods:**

We conducted a comprehensive search in PubMed, PsycINFO, ERIC, and SCOPUS (January 1980–July 2026) using controlled vocabulary and free‐text terms related to SCD and academic performance. We included cross‐sectional studies and one prospective study involving children under 18 years with SCD in LMICs. Eligible studies reported on academic performance outcomes across two measures: academic achievement and grade attainment. Comparator groups were children without SCD. Studies outside LMICs and in languages other than English were excluded. Risk of bias was assessed using the Joanna Briggs Institute Critical Appraisal Checklist for Analytical Cross‐Sectional Studies and Newcastle–Ottawa Scale (NOS). The review was conducted and reported following the PRISMA 2020 guidelines. Owing to the heterogeneity of assessment measures, a meta‐analysis could not be undertaken. Findings were synthesized narratively using the Synthesis Without Meta‐analysis approach.

**Results:**

Of 165 records screened, 11 studies met the inclusion criteria, comprising 1630 participants. Seven of the 11 studies meeting the inclusion criteria had reported poorer academic performance among children with SCD compared to healthy peers, siblings, relatives, or neighborhood controls. Findings varied according to the academic performance measures and analytic approaches employed across studies. Several studies reported poorer academic performance and higher rates of grade repetition among children with SCD compared to controls. Older age and school absenteeism were among the factors most commonly associated with poorer academic outcomes.

**Conclusion:**

Evidence suggests that children with SCD in LMICs may be at increased risk of academic difficulties. Particular attention may be needed for older children with SCD and those with frequent school absenteeism.

**Systematic Review Registration:**

PROSPERO CRD42022200619.

## 1 Introduction

Sickle cell disease (SCD) is a major global health burden, with around 500,000 births each year, approximately 80% of which occur in Sub‐Saharan Africa [1, 2, 3]. SCD refers to a group of inherited hemoglobin (Hb) disorders in which at least one of the two abnormal beta globin genes is HbS, whereas sickle cell anemia (SCA) refers specifically to the homozygous HbSS genotype or the compound heterozygote HbS and Beta° thalassemia (HbS‐β° thalassemia) [4]. SCA is generally considered the most severe form of the disease [5]. Existing neurocognitive research indicates that children with SCD are at increased risk of deficits in domains critical for academic success, such as executive functioning, processing speed, and attention [6, 7, 8]. These disease‐related susceptibilities from severe anemia and the risk of cerebrovascular complications may directly hinder cognitive abilities that are critical for learning [8]. Additional factors associated with poor academic performance among children with SCD include frequent school absenteeism, markers of disease burden, and low Hb levels [3, 9, 10, 11]. In low‐ and middle‐income countries (LMICs), as defined by the World Bank, systemic challenges such as limited access to healthcare, frequent hospitalizations, and socioeconomic status (SES) hardship exacerbate these effects, potentially contributing to cumulative academic challenges [12, 13]. Substantial data from high‐income countries document academic difficulties among children with SCD [9]. There is a notable scarcity of research from LMICs, despite this being the region bearing most of the global disease burden [1].

This systematic review sought to address critical gaps in the literature by examining: (1) the differences in academic performance between children with and without SCD in LMICs; and (2) the factors associated with academic performance outcomes among children with SCD in LMICs.

## 2 Methods

We used the Preferred Reporting Items for Systematic reviews and Meta‐Analyses (PRISMA) 2020 checklist to guide the development, conduct, and reporting of the systematic review [14]. Evidence synthesis was guided by a protocol that was registered in the International Prospective Register of Systematic Reviews (PROSPERO), number CRD42022200619.

### 2.1 Search Strategy

A comprehensive search was conducted across four electronic databases (PubMed, ERIC, PsycINFO, and SCOPUS) and Google Scholar for gray literature. The electronic databases and Google Scholar were searched for studies published between January 1, 1980, and July 14, 2026. The search strategy was developed in collaboration with an experienced librarian skilled in systematic reviews (A. A. K.). A. A. K. and S. K. N. conducted a preliminary search in PubMed to ensure the accuracy and sensitivity of the search strategy in retrieving high‐quality, relevant articles. The finalized PubMed search string was subsequently adapted and used to search for studies in the other databases (Supporting Information S1: Table 1). Medical Subject Headings (MeSH) terms and Boolean operators (OR, AND, NOT) were used to develop the search string. The term “Sickle Cell Trait” was included to maximize the search sensitivity and ensure identification of studies involving different sickle cell genotypes, while studies focusing only on sickle cell trait were excluded during screening. We restricted search terms to the title or abstract and included topic‐specific keywords to maximize relevance and efficiency.

### 2.2 Study Selection

We implemented a rigorous and transparent screening process to minimize bias in study identification and selection. All records were imported into Rayyan (https://www.rayyan.ai), an online tool for systematic review screening, where duplicates were removed. Two reviewers (S. K. N. and S. O.) independently screened all titles and abstracts against the eligibility criteria, with discrepancies resolved by a third reviewer (M. N.) to ensure consistency and objectivity. Full‐text screening was similarly conducted independently by two reviewers (S. K. N. and S. O.), with disagreements again resolved by consensus or by a third reviewer (M. N.). This review approach was employed at all screening stages to reduce selection bias and enhance the reliability of study inclusion.

### 2.3 Eligibility Criteria

We included cross‐sectional and both retrospective and prospective cohort studies published from 1980 through July 14, 2026, that examined academic performance among children with SCD in LMICs. We limited our search to studies published from 1980 onwards to ensure broader and more reliable retrieval of indexed health literature in PubMed and other databases. Eligible participants were children under the age of 18 with confirmed SCD of any genotype, including HbSS, HbSC, HbS‐β^0^ thalassemia, or HbS‐β^+^ thalassemia. Studies were required to report at least one or both of these two academic outcomes: (i) academic achievement measured by standardized test scores, curriculum‐based assessments, three‐term or annual examination averages, subject‐specific scores, class rank, final year percentages, or teacher‐ and parent‐reported school performance; and (ii) grade attainment, measured by whether the child progressed or repeated a grade. Academic achievement outcomes were reported either as continuous measures (e.g., test scores, examination marks, *z*‐scores, or class rank) or categorical classifications (e.g., below average, average, or above‐average performance). We also included studies reporting on factors associated with academic performance in this population. Only studies conducted in countries classified as low‐ or middle‐income were included. LMIC status was determined based on the World Bank classification at the time of the study [13]. We excluded studies conducted outside LMICs, non‐English publications, and those not reporting on relevant academic performance outcomes. Secondary literature, such as commentaries, editorials, and letters, was excluded.

### 2.4 Data Abstraction

Data were abstracted using a standardized data abstraction form developed for this analysis. The tool was piloted in one study to ensure clarity and consistency before full implementation [15]. Extracted data included study characteristics (author, year, country, setting, study design, and study duration), sample details (sample size by group, mean age, age range, proportion of males, and SCD genotype), and comparator descriptions (normal classmates, healthy peers, healthy siblings, and healthy children from the same household, community, or region). Academic performance outcomes were recorded along with their measurement methods (e.g., standardized tests, school examinations, grade retention), statistical results (effect sizes, p‐values, and direction of effects), and factors associated with academic performance when reported. Two reviewers (S. K. N. and S. O.) independently extracted data, and discrepancies were resolved through discussion. If consensus could not be reached, a third reviewer (M. N.) acted as a tie‐breaker. Due to substantial heterogeneity in outcome measures and definitions of academic performance across the included studies, a meta‐analysis could not be conducted. Instead, findings were synthesized narratively using the Synthesis Without Meta‐analysis (SWiM) approach [16].

### 2.5 Risk of Bias Assessment of the Included Studies

The risk of bias of the included cross‐sectional studies (*n* = 10) was assessed using the Joanna Briggs Institute (JBI) Critical Appraisal Checklist for Analytical Cross‐Sectional Studies. The checklist comprises eight items that evaluate the quality of inclusion criteria, measurement of exposures and outcomes, identification of potential confounding factors and strategies to address them, and the appropriateness of statistical analysis. Each item was rated as “Yes,” “No,” “Unclear,” or “Not applicable.” Studies scoring 7–8 were considered having low risk of bias, 5–6 as having moderate risk of bias, and ≤ 4 as having high risk of bias. The risk of bias of the single cohort study was assessed using the Newcastle–Ottawa Quality Assessment Scale for Cohort Studies (NOS), a validated tool for non‐randomized studies. The NOS evaluates three domains: selection of study groups (maximum 4 stars), comparability of groups (maximum 2 stars), and ascertainment of the outcome (maximum 3 stars), for a total of 9 stars. Studies rated 7–9 stars were classified as having low risk of bias, 5–6 stars as having moderate risk of bias, or ≤ 4 stars as having a high risk of bias. Two authors (S. K. N. and S. O.) independently completed the critical appraisals for the selected studies, and a third author (M. N.) cross‐checked the final appraisals. Formal assessment of reporting bias due to missing results was not conducted because the included studies were heterogeneous and the findings were synthesized narratively using the SWiM approach.

## 3 Results

### 3.1 Description of Included Studies

**Figure 1:**
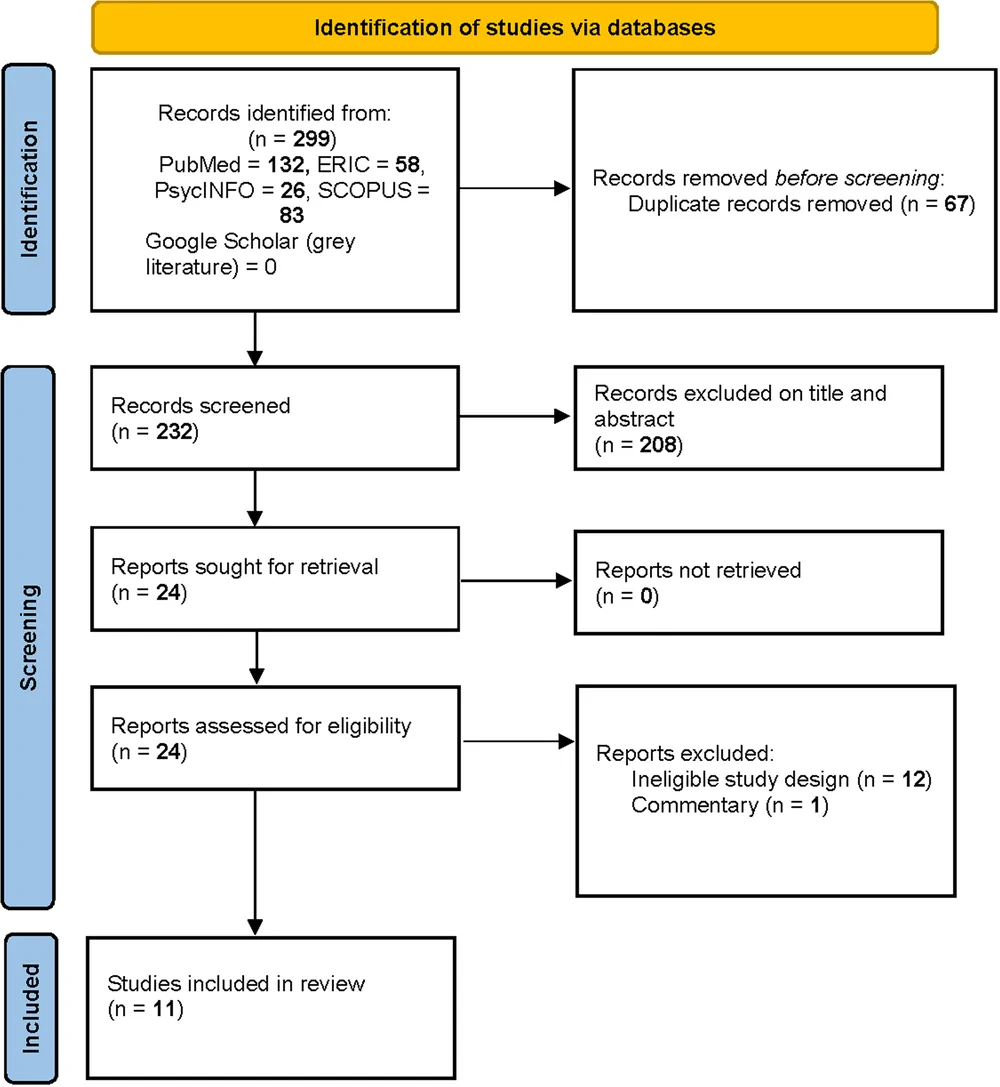
PRISMA flow diagram showing the process of article identification, screening, exclusion, and inclusion in the systematic review. Numbers indicate the count of records at each stage.

A total of 232 records were identified through an initial search of PubMed (*n* = 65), followed by other searches in ERIC (*n* = 58), PsycINFO (*n* = 26), and SCOPUS (*n* = 83). No additional studies were retrieved through hand‐searching or other sources. After de‐duplication, 165 unique records remained for screening. Titles and abstracts were assessed using the predefined eligibility criteria, leading to the exclusion of 141 articles. The full texts of the 24 articles were then reviewed in detail, of which 13 were excluded for not meeting the eligibility criteria. As a result, 11 studies were included in the final review for data extraction. The stages of study inclusion and exclusion are presented using the PRISMA 2020 flow diagram (Figure 1).

The 11 studies comprised data from 1630 participants across 8 LMICs, with most conducted in sub‐Saharan Africa (described below). The included studies comprised primarily cross‐sectional designs (*n* = 10) and one prospective cohort study. Study settings included both hospital and school‐based populations, with participants ranging from early childhood to adolescence (4–17 years). Sample sizes varied, with most studies enrolling between 60 and 313 participants. Academic performance was assessed through various measures, including standardized test scores and school grades. The studies also examined associations between age, gender, grade level, school absenteeism, SES, caregiver education, clinical markers of disease burden, and academic performance outcomes.

Most associations reported in the included studies were derived from unadjusted analyses. Of the 11 studies analyzed, 4 employed multivariable models adjusting for selected socio‐demographic and clinical variables [3, 10, 17, 18]. Disease‐modifying factors such as a history of stroke, silent cerebral infarcts, or hydroxyurea therapy use were generally not included in multivariable analyses of academic performance.

### 3.2 Characteristics of Included Studies

The 11 studies, were published between 2005 and 2025 and conducted in eight countries across Africa (Nigeria [*n* = 4], Benin [*n* = 1], Uganda [*n* = 1], Tanzania [*n* = 1], Morocco [*n* = 1]), the Caribbean (Jamaica [*n* = 1]), South America (Brazil [*n* = 1]), and the Gulf States (Yemen [*n* = 1]) (Table 1).

**Table 1:** Characteristics and academic performance measures of included studies.

References	Country	Setting	Study design	Sample size (cases/controls)	Age range (years)	SCD Genotype	Comparator	Measurement of Academic Performance
Al‐Saqladi [11]	Yemen	Hospital	Cross‐sectional	60/no controls	7–15	SCD (genotype not specified)	Within‐group comparison (mild vs. severe)	Parent‐reported absenteeism, final year grades: Average/above (≥ 60%), Below average (< 60%), and grade repetition
Ezenwosu et al. [19]	Nigeria	Hospital	Cross‐sectional	90/90	5–11	HbSS	Healthy classmates matched for age, sex, and school	Average of three‐term examination scores (categorized as high/average/low) and subject‐specific scores (English, Math, Science, Social Studies)
Ezenwosu et al. [23]	Nigeria	Hospital	Cross‐sectional	90/90	5–11	HbSS	Healthy classmates matched for age, sex, and school	Average of three‐term examination scores and subject‐specific scores (English, Math, Science, Social Studies) categorized as: High (≥ 75%), Average (50–74%), Low (< 50%).
Ikediashi et al. [17]	Benin	Hospital	Cross‐sectional	100/109	6–17	HbSS, HbSC	Pediatric comparisons, healthy siblings	Summative scores on all subjects in one academic year are categorized as: Good (> 14), Average (10–14), Poor (< 10).
King et al. [10]	Jamaica	Hospital	Cross‐sectional	64/Schools' mean test scores	11–13	HbS/β^+^, HbS/β^0^, HbSS	Unaffected peers of similar age, attending the same school, and had taken tests at the same sitting	Grade Six Achievement Test (GSAT) scores in mathematics, language arts, social studies, science, and communication tasks
Matondo et al. [18]	Tanzania	Hospital	Cross‐sectional	233/80	9–15	HbSS	Healthy siblings (without SCD) from the same households	Parent‐reported absenteeism (“more than once”) and class rank (top 10/below top 10)
Naggayi et al. [20]	Uganda	Hospital	Cross‐sectional	68/69	6–12	HbSS, HbS/β^0^	Unaffected age‐appropriate sibling or close relative living in the same neighborhood	Standardized assessment tool: The Wide Range Achievement Test, Fourth Edition (WRAT‐4). Spelling, math computation, word reading, and sentence comprehension *z*‐scores
Ogunfowora et al. [15]	Nigeria	Hospital, School	Cross‐sectional	52/42	6–17	HbSS	School‐age siblings of similar age	School records: absenteeism (days/year), sessional aggregate scores (total sum of scores in all subjects offered during the school year) categorized as: Above average (> 75%), Average (50%–75%), Below average (< 50% and percentage scores in four core subjects (mathematics, English language, integrated science, and social studies)
Olatunya et al. [3]	Nigeria	Hospital	Cross‐sectional	101/101	4–12	HbSS	Healthy classmates matched for age, sex, and SES	Average of the total sum of scores in all subjects offered, categorized as: High (≥ 75%), Average (50%–74%), Low (< 50%).
Raiza et al. [21]	Brazil	University	Cross‐sectional	31/31	8–17	HbSS, HbSC	Healthy siblings, cousins, neighbors, and classmates	Parent‐reported school performance (classified as good, regular, or poor). Structured interviews assessing learning disability reported by the school, class repetition, and school records, percentage scores in mathematics, Portuguese language, natural sciences, and social sciences
Samira et al. [22]	Morocco	School	Prospective cohort	60/66	7–14	—	Healthy children from the same region	Academic performance is categorized as normal schooling and educational backwardness

*Note:* Two studies by Ezenwosu et al. [19, 23] were included: One focused on academic performance outcomes, the other on determinants of academic performance. “—” indicates data not reported.

Abbreviations: HbSC, sickle cell hemoglobin C disease; HbS/β^+^, sickle‐beta plus thalassemia; HbS/β^0^, sickle‐beta zero thalassemia; HbSS, homozygous sickle cell disease; SCD, sickle cell disease; SES, socioeconomic status.

The risk of bias of the included studies ranged from low to moderate. Nine of the cross‐sectional studies were rated as low risk of bias based on the JBI checklist, meeting key criteria such as clear inclusion criteria, valid outcome measurement, and identification of potential confounding factors and strategies to address them (Supporting Information S2: Table 2). Approaches to addressing confounding varied across studies and included matching, exclusion criteria, and, in four studies, multivariable regression models. Several studies relied on bivariate or unadjusted analyses, limiting causal interpretation. Formal statistical adjustment for all relevant socio‐demographic and clinical factors was not consistently performed. The remaining two cross‐sectional studies were rated as moderate risk of bias due to limitations such as unclear measurement of exposure, inadequate identification or management of confounding factors, and less reliable outcome assessment. The cohort study, assessed using the NOS, was also rated as moderate risk of bias (Supporting Information S3: Table 3). Common limitations across studies included single‐center recruitment limiting generalizability, cross‐sectional designs; therefore, change over time could not be assessed, small sample sizes, incomplete adjustment for potential confounders such as SES, parental education, stroke‐related factors, hydroxyurea therapy use, and lack of neuroimaging.

### 3.3 Findings on Academic Performance in Children With SCD in LMICs

#### 3.3.1 Comparison of Academic Performance Between Children With SCD and Controls

Overall, 7 of the 11 studies reported poorer academic outcomes among children with SCD compared to healthy peers, siblings, relatives, or neighborhood controls matched for age, sex, school, or SES (Table 2). Findings were not consistent across the different academic outcome measures. Categorical classifications of academic performance were based on pre‐specified study‐specific thresholds (Table 1), with cut‐points varying across studies.

**Table 2:** Summary of key findings on academic performance among children with sickle cell disease and controls.

References	Outcome measure	SCD^a^	Controls^a^	Statistical test^b^	*p* value	Comment
Al‐Saqladi [11]	Grade repetition	45% (*n* = 27)	NR	NR	NR	A history of grade repetition was reported by 27/60 participants with SCD
	Children‐reported final academic score	48.3% (*n* = 29)	NR	NR	NR	48.3% children reported final academic scores below average
Ezenwosu et al. [19]	Overall academic score (mean of three‐term examination scores)	62.71 ± 19.43%	67.47 ± 16.42%	*t* = −1.78	0.08	No difference between the two groups
	Overall academic score ratings categorized as high/average/low (Proportion of children with low performance)	32.2% (*n* = 29)	16.7% (*n* = 15)	*χ* ^2^ = 5.90	**0.02**	A larger proportion of children with SCA showed significantly lower performance compared to healthy classmates matched for age, sex, and school
	Mean scores of selected key subjects: English language	60.8 ± 23.3	67.7 ± 18.8	*t* = −2.201	**0.03**	The difference between the means was statistically significant for the English Language
Ikediashi et al. [17]	Summative scores on all subjects in one academic year	13.3 ± 2.8	Sibling: 13.1 ± 3.2, Pediatric group: 12.6 ± 2.1	SCD: *t* = 1.29, sibling: *t* = 0.33, pediatric group: *t* = 1.75	Pediatric group: 0.08, SCD: 0.20, sibling: 0.74	No significant differences were observed between the patient group (SCD) and both comparison groups
King et al. [10]	GSAT mathematics, language arts, social studies, science, and communication task *z*‐scores	NR	NR	Language: MD = −0.28 (95% CI: −0.55 to −0.003), Math: MD = −0.29 (95% CI: −0.55 to −0.02), Science: MD = −0.36 (95% CI: −0.63 to −0.09), Social studies: MD = −0.32 (95% CI: −0.58 to −0.05)	Language: **0.04,** Math: **0.03,** Science: **0.009,** Social studies: **0.02**	The mean difference in *z*‐scores between children with SCD and their peers was statistically significant in all subject areas except for the communication task
Matondo et al. [18]	Parent reported class rank (top 10/below top 10)	NR	NR	NR	NR	Cases were more in the “below top 10” class position compared to normal siblings
Naggayi et al. [20]	WRAT‐4 spelling, math computation, word reading, and sentence comprehension *z*‐scores (Mathematical computation)	Median [IQR] −0.47 [−1.11 to 0.08]	Median [IQR] −0.02 [−0.46 to 0.61]	NR^c^	**0.001**	Children with SCA had significantly poorer proficiency in mathematical computation compared to siblings
Ogunfowora et al. [15]	Total sum of scores in all subjects offered during the school year (Sessional aggregate score)	Range 26.5%–90.2%, mean 62.6 ± 15.3%	Range 34.1%–88.1%, mean of 64.8 ± 14.1%	*t* = 0.510	> 0.50	No significant difference between the mean aggregate scores of children with SCA and siblings
	Grading of academic performance based on SAS is categorized as above‐average (SAS > 75%), average (SAS 50%–75%), and below‐average (SAS < 50%)	Above average: *n* = 12 (23%), average: *n* = 27 (51.9%), below‐average: *n* = 13 (25%)	Above‐average: *n* = 8 (19%), average: *n* = 32 (76%), below‐average: *n* = 2 (4.8%)	Above‐average: *χ* ^2^ = 0.23, average: *χ* ^2^ = 5.85, below‐average: *χ* ^2^ = 5.67	Above‐average: 0.64, average: **0.02**, below‐average: **0.02**	A higher proportion of children with SCA were average or below‐average achievers compared to siblings. No significant difference in the above‐average category
	Percentage scores in four core subjects (Mathematics, English language, integrated science, and social studies)	Mathematics: 59.2%, English: 64%, Science: 63.9%, Social studies: 63.8%	Mathematics: 61.9%, English: 64.4%, Science: 65.8%, Social studies: 64.8%	Mathematics: *t* = 1.113, English: *t* = 0.106, Science: *t* = 0.76, Social studies: *t* = 0.412	Mathematics: 0.29, English: 0.73, Science: 0.42, Social studies: 0.58	No difference between the two groups in the four core subjects
Olatunya et al. [3]	Average of the total sum of scores in all subjects	59.0 ± 11.1	60.6 ± 9.8	*t* = 1.139	0.26	No difference between the two groups
Raiza et al. [21]	Parent‐reported school performance (classified as good, regular, or poor)	Good: 45.2%, regular: 29.0%, poor: 25.8%	Good: 67.7%, regular: 19.4%, poor: 12.9%	NR	0.19	No difference between the two groups
Samira et al. [22]	Grade repetition percentages	Repeaters: 60%, non‐repeaters: 40%	Repeaters: 27.27%, non‐repeaters: 72.73%	*χ* ^2^ = 13.75	**< 0.001**	Children with SCD had significantly higher repetition rates

*Note:* Bold values indicate statistical findings (*p* < 0.05).

Abbreviations: GSAT, Grade Six Achievement Test; NR, not reported; SAS, sessional aggregate score; WRAT‐4, Wide Range Achievement Test.

^a^ Descriptive (mean ± SD, median [IQR], %).

^b^ Inferential (e.g., *t*‐test, *χ* ^2^, mean difference [MD] with 95% CI, Wilcoxon rank‐sum test) as reported by the original studies.

^c^ Wilcoxon rank‐sum (Mann–Whitney) was used to compare groups.

Two studies from Nigeria assessed academic achievement using both continuous measures (mean academic scores) and categorical classifications of performance, representing distinct analytic approaches. Ezenwosu et al. [19] classified academic performance as high (≥ 75%), average (50%–74%), or low (< 50%), while Ogunfowora et al. [15] classified performance as above average (> 75%), average (50%–75%), or below average (< 50%). Using mean academic scores, Ezenwosu et al. [19] reported lower academic scores among children with SCA compared to healthy classmates matched for age, sex and school (62.7 ± 19.4% vs. 67.5 ± 16.4%), but the difference between the two groups was not significant (*p* = 0.08). Similarly, Ogunfowora et al. [15] found no significant difference in mean academic achievement between children with SCA and their age‐matched siblings (*t* = 0.510, *p* > 0.50). However, further categorical analyses in both studies demonstrated a higher proportion of children with SCA in lower‐performing categories compared to controls. In the study by Ezenwosu et al., 32.2% of children with SCA were classified as low performers compared with 16.7% of healthy classmates (*χ* ^2^ = 5.90, *p* = 0.02). Similarly, Ogunfowora et al. reported that 25.0% of children with SCA were below‐average achievers compared with 4.8% of their siblings (*χ* ^2^ = 5.67, *p* = 0.02) (Table 2).

Four studies evaluated subject‐specific academic achievement. Three reported poorer performance among children with SCD, particularly in mathematics and language‐based subjects, whereas one study found no significant differences between the groups. King et al. [10], reported that children with SCD had significantly lower mean Grade Six Achievement Test (GSAT) *z*‐scores than their healthy school peers in mathematics (MD = −0.29, 95% CI: −0.55 to −0.02), language arts (MD = −0.28, 95% CI: −0.55 to −0.003), science (MD = −0.36, 95% CI: −0.63 to −0.09), and social studies (MD = −0.32, 95% CI: −0.58 to −0.05), while no significant difference was observed for the communication task. In the Ugandan study, Naggayi et al. [20] reported poorer mathematical computation among children with SCA than age‐matched sibling controls (median [IQR]: −0.47 [−1.11 to 0.08] vs. −0.02 [−0.46 to 0.61], *p* = 0.001). Ezenwosu and colleagues also found poorer English language performance among children with SCA compared to healthy classmates [19] (Table 2). On the contrary, Ogunfowora et al. [15] observed no significant difference between children with SCA and their siblings across the four core subjects.

In contrast, three studies found no significant differences in academic achievement scores between children with SCD and comparison groups. Olatunya et al. [3] in Nigeria reported comparable mean academic scores in all subjects between children with SCA and healthy peers. Similarly, Ikediashi et al. [17] observed no differences in mean summative scores in all subjects among children with SCD, siblings, and pediatric controls in Benin. The study from Brazil also found no significant differences in parent‐reported school performance between children with SCD and their healthy counterparts [21].

Only two studies reported grade attainment outcomes, and only one provided formal statistical comparisons between children with SCD and controls [22]. In the Moroccan cohort, grade attainment differed significantly between groups, with grade repetition rates markedly higher in children with SCD than in healthy peers (60% vs. 27.3%, *p* < 0.001) [22]. Al‐Saqladi [11] reported that 27 of 60 children with SCD (45%) had a history of grade repetition based on parent reports.

#### 3.3.2 Factors Associated With Academic Performance in Children With SCD

Older age emerged as the factor most commonly associated with poorer academic performance among children with SCD, although the strength and significance of this association varied across studies (Table 3). Across multiple studies, older children tended to have lower academic achievement and higher rates of grade repetition than younger children [3, 11, 17, 20, 23]. In Nigeria, Ezenwonsu et al. [23] reported a negative correlation between older age and overall academic scores. Similar findings were reported in Benin [17], where older age remained significantly associated with academic performance after adjustment for educational level and maternal education in the multivariable model (*β* = −0.263, *p* = 0.05). Olatunya et al. [3] found that poor academic achievement was more common among children older than 10 years in bivariate analyses. However, the association was not significant in the multivariable model. Al‐Saqladi [11] found that older age was positively correlated with grade repetition and school absenteeism. In Uganda, Naggayi et al. [20] observed a negative association between age and spelling performance, with older children tending to have poorer spelling *z*‐scores (*ρ* = −0.27, *p* = 0.02); however, the association was no longer statistically significant after correction for multiple comparisons.

**Table 3:** Factors associated with academic performance among children with SCD in LMICs.

Factor	Studies	Academic measure	Association^a^	*p* value	Direction of association
Age	Al‐Saqladi [11]	Grade repetition	*r* = 0.32	0.01	Older age was associated with a higher rate of grade repetition
	Ezenwosu et al. [23]	Overall academic scores	*r* = −0.412	< 0.001	Older age associated with poorer performance
	Ikediashi et al. [17]	Overall academic scores	Univariable: *β* = −0.309 Multivariable: *β* = −0.263	< 0.001 0.05	Older age remained independently associated with poorer academic scores in the multivariable model
	Naggayi et al. [20]	WRAT‐4 spelling *z*‐scores	*ρ* = −0.27	0.02 (Not significant; threshold *p* < 0.0125)	A negative correlation was observed between older age and spelling; the association was lost after correction for multiple comparisons
	Olatunya et al. [3]	Average of the total sum of scores in all subjects	Age > 10 years: *χ* ^2^ = 5.164	0.02	A higher proportion of children with poor academic performance were aged > 10 years; the association was not retained in the multivariable model
Sex	King et al. [10]	GSAT mathematics, language arts, social studies, science, and communication task *z*‐scores	Language: MD = −0.27 (95% CI: −0.64 to 0.09), Math: MD = −0.46 (95% CI: −0.83 to −0.09), Communication task: MD = −0.41 (95% CI: −0.82 to −0.01), Science: MD = −0.50 (95% CI: −0.87 to −0.13), Social studies: MD = −0.50 (95% CI: −0.85 to −0.16)	Language: 0.14, Math: 0.02, Communication task: 0.05, Science: 0.01, Social studies: 0.01	Males with SCD scored significantly lower than females across all subject areas except language
Hemoglobin level	King et al. [10]	GSAT mathematics, language arts, social studies, science, and communication task *z*‐scores	Language: *β* = 0.29 (95% CI: 0.054 to 0.532), Math: *β* = 0.25 (95% CI: 0.008 to 0.490), Communication task: *β* = 0.25 (95% CI: 0.003 to 0.50), Social studies: *β* = 0.30 (95% CI: 0.07 to 0.541)	Language: 0.02, Math: 0.04, Communication task: 0.05, Social studies: 0.01	Higher Hb levels were significantly associated with better academic performance, with each 1‐unit increase in Hb corresponding to a 0.22–0.27 standard deviation improvement in subject score rank
Disease severity	Al‐Saqladi [11]	Grade retention	Mild versus severe: *n* = 1 (4.7%) versus *n* = 26 (66.6%)	< 0.001	Children with a history of grade retention had significantly higher disease severity than those without
	Al‐Saqladi [11]	Final academic score in a year	Mild versus severe: *n* = 6 (28.5%) versus 23 (58.9%)	< 0.05	Children with higher disease severity reported final academic scores below average compared to those with lower disease severity
Intelligence	Ezenwosu et al. [23]	Overall academic scores	*r* = 0.394	< 0.001	Overall academic scores showed a significant positive correlation with intelligence scores
SNHL	Raiza et al. [21]	School performance	SNHL: *n* = 5 (62.5%) versus without SNHL: *n* = 3 (13%)	0.02	Children with SNHL exhibited poorer school performance compared to those without SNHL
School absenteeism	Al‐Saqladi [11]	Grade retention	median [IQR] = 52 [40–110] days, versus those with no history of grade retention, median [IQR] = 14 [7–28] days	< 0.001	Children with grade retention missed more school days than those without grade retention
	Olatunya et al. [3]	Average of the total sum of scores in all subjects	Bivariate: 87.5% versus 36.4%; *χ* ^2^ = 19.155 Multivariable: OR = 15.71 (95% CI: 5.93–41.66)	< 0.001	School absenteeism (> 1 week) remained independently associated with poorer academic performance in the multivariable model
Academic class/Education level	Ikediashi et al. [17]	Overall academic scores	Univariable: *β* = −1.528 Multivariable: *β* = 0.201	0.006 0.81	Children in the primary education level had better academic performance compared to those in the secondary education level in univariate analysis; the association was not retained after multivariable adjustment.
	Olatunya et al. [3]	Average of the total sum of scores in all subjects	Poor school performance: 75.0% versus normal school performance 45.5%; *χ* ^2^ = 6.405	0.01	Higher classes (primary 4–6) are significantly associated with poorer school performance in bivariate analyses
Maternal education	Ikediashi et al. [17]	Overall academic scores	*Univariable: β* = 2.083 Multivariable: *β* = 1.376	0.009 0.07	Tertiary maternal education is positively associated with academic performance in univariate analysis; the association was not retained after multivariable adjustment
Socioeconomic status	Ezenwosu et al. [23]	Overall academic scores	*r* = 0.313	0.003	Lower socioeconomic status was associated with poorer overall academic scores in unadjusted analyses

*Note:* Between‐group comparisons (SCD vs. controls) were excluded from this table. Associations were derived from both unadjusted and adjusted analyses. Details regarding covariates included in multivariable models are described in the text.

Abbreviations: GSAT, Grade Six Achievement Test; SNHL, Sensorineural hearing loss; WRAT‐4, Wide Range Achievement Test.

^a^ Values reported include descriptive statistics (median [IQR], %) and group comparisons (mean differences [MD] with 95% CI) as well as measures of association (correlation coefficients *r*, *ρ*; regression coefficients *β*; chi‐square or *t*‐test statistics), as reported in the original studies.

School absenteeism was also commonly associated with poorer academic performance in children with SCD across multiple studies. In Yemen, school absences exceeding 20 days were reported in 60% of children with SCD. Children with a history of grade retention had substantially more school absences than those without grade retention (median [IQR]: 52 [40–110] vs. 14 [7–28] days; *p* < 0.001) [11]. Similarly, in Nigeria, school absenteeism exceeding 1 week was significantly associated with poor academic performance and remained independently associated with it in multivariable analysis (OR = 15.71, 95% CI: 5.93–41.66, *p* < 0.001) [3]. However, those findings were not uniform across studies. Ogunfowora et al. [15] reported more missed school days among children with SCA than their siblings but found no association between school absenteeism and academic performance. Likewise, Ezenwosu et al. [23] found no association between school absenteeism and academic performance.

Educational level was also associated with academic performance. In Benin, children in secondary school demonstrated poorer academic performance than those in primary school in univariate analysis (*β* = −0.528, *p* = 0.006); however, the association was not retained after adjustment for age and maternal education in the multivariable model [17]. Similarly, Olatunya et al. [3] found that students in upper primary classes had poorer academic performance than those in lower primary grades in bivariate analyses. However, this association was not retained in the multivariable model. Naggayi et al. [20] did not observe a clear association between grade level and academic outcomes; however, interpretation was limited by incomplete grade level data.

In the Yemeni study, children classified as having more severe disease had higher grade repetition rates (66.6% vs. 4.7%, *p* < 0.001) and a higher proportion of below‐average annual academic scores (58.9% vs. 28.5%, *p* < 0.05) than those with milder disease [11]. Severity was assessed using a composite index of disease severity based on clinical morbidity indicators, including pain crises, hospitalizations, blood transfusions, disease‐related complications, and degree of anemia (Hb levels). In the Jamaican cohort, higher Hb levels were associated with better academic outcomes, with each 1‐unit increase in Hb predicting approximately a 0.22–0.27 SD increase in subject score rank after adjustment for caregiver socio‐demographic factors (education, employment status, and living environment) [10]. Sensorineural hearing loss was also associated with poorer academic outcomes in the Brazilian study, with affected children more likely to have poor school performance than those without SNHL (62.5% vs. 13.0%, *p* = 0.02) [21].

Furthermore, Intelligence quotient (IQ) was associated with academic performance. Ezenwosu et al. [23] reported a positive correlation between IQ scores and overall academic performance among Nigerian children with SCA (*r* = 0.394, *p* < 0.001) [23].

Sex differences were reported in the Jamaican cohort, where males with SCD scored significantly lower than females across most subject areas except in language arts [10]. However, several other studies found no association between sex and academic performance [3, 11, 17, 20].

Evidence regarding SES and parental factors was mixed. In Nigeria, Ezenwosu et al. [23] found that children from lower SES backgrounds tended to have poorer academic performance. In Benin, tertiary‐level maternal education, a proxy measure of higher SES, was associated with higher academic performance in univariate analysis (*β* = 2.083, *p* = 0.009); however, the association was not retained in the multivariable model after adjustment for age and educational level. Contrary to those findings, Olatunya et al. [3] and Naggayi et al. [20] did not find significant associations between SES or maternal education and academic performance, respectively. Similarly, the Jamaican study found no association between caregiver socio‐demographic factors (education, employment status, and living environment) and academic performance after adjustment for Hb level [10].

## 4 Discussion

This is the first comprehensive review to evaluate academic performance among children with SCD in LMICs. The review synthesized available evidence on academic performance in this population and explored associated determinants. The findings of this review indicate that children with SCD in LMICs may be at increased risk of poorer academic performance compared to children without SCD. These findings conform with broader literature from high‐income settings, where children with SCD have also been reported to experience poorer academic performance compared to healthy siblings, classmates, and peers [7, 9, 24]. Findings in this review varied according to the academic outcome measure and analytic approach used. In some studies, categorical classifications of academic performance identified differences between children with SCD and controls that were not observed when continuous measures of academic achievement were compared.

Among studies that assessed specific academic subjects, poorer performance was most commonly reported in mathematics and language‐based domains [10, 19, 20]. These results corroborate research from the United States showing that students with SCD tend to perform below average in reading, mathematics, and spelling [9]. Another recent U.S. study by Heitzer et al. [25] using statewide academic and medical records found that students with SCD had lower English proficiency compared to healthy children, highlighting the potential impact of SCD on language‐based academic outcomes. This finding is biologically plausible given that skills in these subject areas rely on higher‐level performance, including working memory, attention, and language processing domains, which are affected in sickle cell‐related neurocognitive impairment [8, 26].

Evidence on grade repetition in children with SCD in LMICs was limited, but available findings suggest challenges in academic progression [11, 22]. U.S.‐based evidence indicates that grade retention is common among students with SCD [27], including an increased likelihood of retention in ninth grade compared to healthy peers [25]. In LMICs, where compensatory interventions such as remedial support, individualized education plans, and cognitive rehabilitation are limited, the academic consequences of grade repetition may be more pronounced. The scarcity of data on grade repetition in LMICs highlights an important gap in the literature on academic attainment among children with SCD and underscores the need for further research.

Older age was commonly associated with poorer academic performance, consistent with wider literature from the United States [9]. Moreover, children with SCD in the lower grades performed better than those in the upper grades. This association may reflect the cumulative impact of SCD‐related complications on learning as children progress through school [3, 17, 28]. As children with SCD grow older, the cumulative effects of frequent school absenteeism and disease‐related complications, including cerebrovascular risk, may contribute to academic difficulties [29, 30, 31, 32, 33].

School absenteeism was reported as an important factor associated with academic performance outcomes among children with SCD in LMICs. In one Nigerian study, absenteeism remained independently associated with poor academic performance after multivariable adjustment [3]. Frequent absences due to pain crises, hospitalizations, or clinic visits disrupt learning continuity and classroom engagement [34]. Over time, these interruptions may lead to gaps in knowledge, lower academic achievement, and grade repetition [9]. Evidence from the United States similarly indicates that school absenteeism is linked to poorer educational outcomes among children with SCD despite greater access to medical care [34].

Sex was generally not associated with academic performance in children with SCD, with most studies in our review reporting no significant differences between males and females [3, 11, 17, 20]. However, one study in Jamaica found that male children with SCD performed poorer than their female counterparts [10]. This finding may reflect broader contextual trends in the Jamaican educational system, where girls outperform boys in examinations and overall academic performance [35]. Future studies should continue to explore the role of sex in academic performance among children with SCD.

While only one study reported associations between disease severity and academic outcomes, possibly there is a heavier clinical burden that may adversely affect academic performance among children with SCD in low‐resource settings [11]. Similar patterns have been documented in the United States, where greater disease‐related illness was linked to increased school absenteeism, which in turn undermines academic performance [36].

Furthermore, a higher Hb level was positively associated with academic performance, reinforcing the link between key biological disease markers and school performance [10]. Although this association was reported in only one study, it aligns with existing evidence linking chronic anemia in SCD to adverse neurocognitive outcomes. Lower hemoglobin may impair cerebral oxygen delivery, potentially compromising neurocognitive functions that are critical for learning [37, 38, 39].

The association between lower intellectual ability and poorer academic performance among Nigerian children with SCD aligns with reports from various studies in the United States, which have shown that intellectual functioning is strongly associated with reading and math performance [9]. Similarly, Schatz and colleagues reported that cognitive ability was associated with poor academic outcomes among children in the United States [36]. Collectively, these results suggest that neurocognitive deficits common in SCD, such as slowed processing speed, deficits in working memory, and executive functioning, may affect learning in the classroom [8].

Moreover, hearing loss, which has been reported among children with SCD, may also lead to poorer cognition and thus contribute to educational challenges [21]. In this population, sensorineural hearing loss is common and likely results from cochlear microvascular injury or disruption of auditory processing necessary for classroom learning [40, 41]. Hearing impairment is well known to affect word recognition, spelling, and language skills, thus leading to grade repetition or the need for special education [42].

### 4.1 Limitations

First, the geographic concentration of studies in Nigeria may limit the generalizability of the findings. However, this potential bias is mitigated by Nigeria having the largest number of children affected by SCD worldwide [1]. Secondly, methodological heterogeneity in academic outcome measures prevented quantitative synthesis for a meta‐analysis and likely contributed to inconsistent findings. The paucity of neuroimaging data in this review represents a notable gap, as silent cerebral infarcts are known to impact cognitive function and, consequently, academic performance in SCD. Additionally, most studies relied on unadjusted analyses or adjusted for a limited number of socio‐demographic and clinical variables. Strokes, silent cerebral infarcts, and hydroxyurea therapy use were rarely assessed or controlled for, limiting interpretation of observed associations and raising the possibility of confounding. Clinical markers of disease burden were assessed differently across studies, limiting comparisons of findings related to clinical disease factors and academic performance. Lastly, most included studies were cross‐sectional, limiting causal inferences. Future research should prioritize longitudinal designs, standardized academic assessments, and integration of neuroimaging and neurocognitive data to clarify the pathways linking disease burden to academic functioning.

### 4.2 Implications

This review highlights the academic challenges faced by children with SCD in LMICs, where disease burden is highest, yet academic outcomes remain understudied. Although findings varied across studies, the available evidence suggests that children with SCD experience educational challenges that warrant attention from educators and health care providers in LMICs.

These findings underscore the importance of recognizing the effects of disease‐related complications and school absenteeism on learning among children with SCD in LMICs. Future research should employ longitudinal designs using standardized academic performance assessments across diverse settings. Intervention studies are needed to evaluate whether strategies that address school‐based challenges, such as catch‐up tutoring for children with frequent absences, as well as improved access to disease‐modifying therapies and standard SCD care, can improve academic outcomes among children with SCD in LMICs.

## 5 Conclusion

Evidence from this review suggests that children with SCD in LMICs experience important educational challenges. Older age and school absenteeism may place some children at greater risk of academic difficulties. Future research should evaluate whether disease‐modifying therapies and standard SCD care can reduce school absenteeism and other barriers to learning, and improve academic outcomes among children with SCD in LMICs.

## Author Contributions

S.K.N. conceived the study, authored, and oversaw the execution of the systematic review. P.B., A.A.K., D.A., N.S.G., and R.I. provided important intellectual content, subject‐specific guidance, and manuscript editing. S.O. and M.N. provided conceptual and methodological guidance throughout the review and contributed to manuscript editing. All authors have read and approved the final version of the manuscript. S.K.N., the corresponding author, had full access to all data in this study and takes complete responsibility for the integrity and accuracy of the data analysis.

## Ethics Statement

Ethical approval and informed consent were not required for this study because it was a systematic review of previously published studies and did not involve the collection of primary data from human participants or the use of identifiable personal information.

## Conflicts of Interest

The authors declare no conflicts of interest.

## Transparency Statement

The lead author and corresponding author (Shubaya K. Naggayi) affirms that this manuscript is an honest, accurate, and transparent account of the study being reported; that no important aspects of the study have been omitted; and that any discrepancies from the study as planned and registered have been explained.

## Supporting information


Supporting File 1

Supporting File 2

Supporting File 3

## Data Availability

The authors confirm that all data supporting the findings of this study are available within the article and its supporting materials. All data analyzed in this systematic review were extracted from previously published studies, which are cited in the reference list.
