# Supplementary material for: Academic Performance Among Children With Sickle Cell Disease in Low‐ and Middle‐Income Countries: A Systematic Review
Source: Health Sci Rep. 2026 Sep 3;9(9):e73150. doi: 10.1002/hsr2.73150 (PMC13539348; doi:10.1002/hsr2.73150)
Supplement: Supplementary file 1 [file HSR2-9-e73150-s001.docx]

Supplementary Table 2. Risk of bias assessment of analytical cross-sectional studies using JBI critical appraisal checklist.

| Authors (Year) | Were the criteria for inclusion in the sample clearly defined? | Were the study subjects and the setting described in detail? | Was the exposure measured in a valid and reliable way? | Were objective, standard criteria used for measurement of the condition? | Were confounding factors identified? | Were strategies to deal with confounding factors stated? | Were the outcomes measured in a valid and reliable way? | Was appropriate statistical analysis used? | Overall score† | Risk of Bias rating |
| --- | --- | --- | --- | --- | --- | --- | --- | --- | --- | --- |
| Al-Saqladi et al. (2016) | Yes | Yes | Yes | Yes | No | No | No | Yes | 5 | Moderate risk of bias |
| Ezenwosu et al. (2013) | Yes | Yes | Yes | Yes | Yes | Yes | Yes | Yes | 8 | Low risk of bias |
| Ezenwosu et al. (2013b) | Yes | Yes | Yes | Unclear | Yes | Yes | Yes | Yes | 7 | Low risk of bias |
| Ikediashi et al. (2025) | Yes | Yes | Unclear | No | Yes | Yes | Yes | Yes | 6 | Moderate risk of bias |
| King et al. (2023) | Yes | Yes | Yes | Unclear | Yes | Yes | Yes | Yes | 7 | Low risk of bias |
| Matondo et al. (2020) | Yes | Yes | Yes | Yes | Yes | Yes | Yes | Yes | 8 | Low risk of bias |
| Naggayi et al. (2025) | Yes | Yes | Yes | Yes | Yes | Yes | Yes | Yes | 8 | Low risk of bias |
| Ogunfowora et al. (2005) | Yes | Yes | Yes | Unclear | Yes | Yes | Yes | Yes | 7 | Low risk of bias |
| Olatunya et al. (2017) | Yes | Yes | Yes | Unclear | Yes | Yes | Yes | Yes | 7 | Low risk of bias |
| Raiza et al. (2022) | Yes | Yes | Yes | Yes | Yes | Yes | No | Yes | 7 | Low risk of bias |

†The JBI checklist overall scores were interpreted as follows: 7–8 = low risk of bias, 5–6 = moderate risk of bias, ≤4 = high risk of bias.
