# Supplementary material for: Academic Performance Among Children With Sickle Cell Disease in Low‐ and Middle‐Income Countries: A Systematic Review
Source: Health Sci Rep. 2026 Sep 3;9(9):e73150. doi: 10.1002/hsr2.73150 (PMC13539348; doi:10.1002/hsr2.73150)
Supplement: Supplementary file 2 [file HSR2-9-e73150-s002.docx]

Supplementary Table 1. PubMed Search Strategy for Identifying Studies on Academic Performance in Children with Sickle Cell Disease in LMICs

| 1 | (Academic performance* OR Academic Test Performance* OR Academic Test Score* OR Educational Test Score* OR Educational Test Performance* OR Academic Success* OR Academic Achievement* OR Academic Attainment).tiab.tw |
| --- | --- |
| 2 | (Child* OR Minor OR Adolescents OR Adolescence OR Teen* OR Youth* OR Female Adolescent* OR Male Adolescent* OR Infant* OR Preschool Child* OR School Age OR school going).tiab.tw |
| 3 | 1 AND 2 |
| 4 | (Sickle Cell OR Sickle Cell Anemia* OR Hemoglobin S Disease OR Hemoglobin S Diseases OR Sickle Cell Disorder* OR HbS Disease OR Sickle Cell Disease OR Hemoglobin SC Diseases OR Sickle Cell Hemoglobin C Disease OR SC Disease OR SC Diseases OR Sickle Cell Trait* OR Sickle Cell Anaemia*).tiab.tw |
| 5 | 3 AND 4 |

A librarian (AAK) developed the initial search strategy in PubMed, focusing on key terms related to sickle cell disease, academic performance, academic achievement, and children. Both MeSH terms and free-text keywords were used to include all relevant synonyms. The search was conducted across the title, abstract, and keyword fields. The strategy was adapted for other databases.
