# Supplementary material for: Academic Performance Among Children With Sickle Cell Disease in Low‐ and Middle‐Income Countries: A Systematic Review
Source: Health Sci Rep. 2026 Sep 3;9(9):e73150. doi: 10.1002/hsr2.73150 (PMC13539348; doi:10.1002/hsr2.73150)
Supplement: Supplementary file 3 [file HSR2-9-e73150-s003.docx]

Supplementary Table 3. Risk of bias assessment using the Newcastle-Ottawa Quality Assessment Scale for cohort studies

| Authors (Year) | Selection (Max 4) | Comparability (Max 2) | Outcome (Max 3) | Overall score† | Risk of bias rating |
| --- | --- | --- | --- | --- | --- |
| Samira et al. (2019) | 3 | 1 | 2 | 6 | Moderate risk of bias |

†The NOS overall scores were interpreted as follows: 7–9 = low risk of bias, 5–6 = moderate risk of bias, ≤4 = high risk of bias.
